# Is Familial Mediterranean Fever a clinical diagnosis? Results of a field survey

**DOI:** 10.1186/1546-0096-13-S1-P126

**Published:** 2015-09-28

**Authors:** H Ozdogan, S Ugurlu, G Hatemi, E Ozgun, G Can, G Ozgon, B Ergezen, Z Study Group

**Affiliations:** 1 Cerrahpasa Medical Faculty, University of Istanbul, Division of Rheumatology, Department of Internal Medicine, Istanbul, Turkey

## Background

It has been reported that FMF was more prevalent among Turkish people whose parental origin came from central Anatolia.

## Objectives

To determine the prevalence of clinically diagnosed FMF blind to the results of mutation analysis and to assess the frequency and distribution of MEFV mutations, in Sivas, Zara.

## Methods

15906 people live in Zara, a small town of Sivas. To detect a prevalence of 0.6% with an error of 0.35% and 95% CI, 1673 people were to be screened. One parent from each house (index person) was directly interviewed and also questioned for the family members (FM) of the household with two standard questionnaires. Subjects were revisited by a group of rheumatologists for 3 times. Finally they were classified clinically as definite, probable and possible FMF, blind to genetic analysis. Blood was drawn from every fifth index case (n=336) to analyze MEFV-mutations by sequencing and from the definite and suspected cases of FMF (104/121, 86%).

## Results

1727 inhabitants were interviewed directly (index population). (M:F=176:1551, mean age: 40.2±11.4). Information concerning the 4591 family members was obtained via the index person (M:F=2939:1652, mean age: 33.5±19.6). According to the results of the first visit, 384/6318 (165 index, 219 FM) had suspected FMF. In the second visit 368/384 (95.8%) were contacted. A final diagnosis based on clinical criteria, was obtained after the third visit (121/368, 47 index, 74 family members). Among 121 patients 68 were considered as definite (68/6318;1.07%), 21 as probable (21/6318;0.34%) and 32 as possible FMF (32/6318;0.50%). The results of the mutation analysis were unblinded only after this classification was finalized. The distribution of patients carrying at least one exon 10 mutation were 32 of the 60 tested (53.3%) in the definite, 5 out of 19 (26.3%) in the probable, and 11 of the 25 (44.0%) in the possible group. In the general Zara population, 60 of the 336 tested (17.8%) carried at least one exon 10 mutation.

## Conclusions

The minimal prevalence of definite clinical FMF in Zara was estimated as 1.07%. Only half of the patient group, as a whole, carried at least one exon 10 mutation. This was significantly different from the carrier rate of exon 10 mutations in the general population of Zara (1:6). However, even in a small population like this, MEFV gene mutations do not seem to be the only explanation why clinical FMF is so prevalent among people of Zara.

